# Actin cytoskeleton remodeling at the cancer cell side of the immunological synapse: good, bad, or both?

**DOI:** 10.3389/fimmu.2023.1276602

**Published:** 2023-10-05

**Authors:** Elena Ockfen, Liza Filali, Diogo Pereira Fernandes, Céline Hoffmann, Clément Thomas

**Affiliations:** ^1^Cytoskeleton and Cancer Progression, Department of Cancer Research, Luxembourg Institute of Health, Luxembourg, Luxembourg; ^2^Faculty of Science, Technology and Medicine University of Luxembourg, Esch-sur-Alzette, Luxembourg

**Keywords:** actin cytoskeleton, cancer, cytotoxic lymphocytes, cytotoxic T cells, immunological synapse, natural killer cells

## Abstract

Cytotoxic lymphocytes (CLs), specifically cytotoxic T lymphocytes and natural killer cells, are indispensable guardians of the immune system and orchestrate the recognition and elimination of cancer cells. Upon encountering a cancer cell, CLs establish a specialized cellular junction, known as the immunological synapse that stands as a pivotal determinant for effective cell killing. Extensive research has focused on the presynaptic side of the immunological synapse and elucidated the multiple functions of the CL actin cytoskeleton in synapse formation, organization, regulatory signaling, and lytic activity. In contrast, the postsynaptic (cancer cell) counterpart has remained relatively unexplored. Nevertheless, both indirect and direct evidence has begun to illuminate the significant and profound consequences of cytoskeletal changes within cancer cells on the outcome of the lytic immunological synapse. Here, we explore the understudied role of the cancer cell actin cytoskeleton in modulating the immune response within the immunological synapse. We shed light on the intricate interplay between actin dynamics and the evasion mechanisms employed by cancer cells, thus providing potential routes for future research and envisioning therapeutic interventions targeting the postsynaptic side of the immunological synapse in the realm of cancer immunotherapy. This review article highlights the importance of actin dynamics within the immunological synapse between cytotoxic lymphocytes and cancer cells focusing on the less-explored postsynaptic side of the synapse. It presents emerging evidence that actin dynamics in cancer cells can critically influence the outcome of cytotoxic lymphocyte interactions with cancer cells.

## Introduction

The anti-tumor immune response is a complex process involving a coordinated interplay between various immune cells. At the forefront of this response are cytotoxic lymphocytes (CLs), mainly natural killer (NK) cells and CD8+ cytotoxic lymphocytes (CTLs), that are essential for identifying and eliminating cancer cells. NK cells are the frontline defense against cancer cells due to their innate capability to rapidly recognize and kill target cells without prior sensitization or the need for clonal expansion (1). They play a significant role in the control of early tumors and metastasis (2, 3) while they are typically less abundant than CTLs in the tumor microenvironment (TME) of more advanced tumors (4, 5). Presently, CTLs are recognized as the most potent effectors in the anti-tumor immune response and represent the primary focus of current cancer immunotherapies involving immune-checkpoint inhibitors and genetic modification of receptors (6, 7).

NK cells have a large repertoire of surface inhibitory and activating receptors, which enable them to differentiate between healthy and diseased cells (8–11). Most host’s healthy cells express self-major histocompatibility complex class I (MHCI) molecules that act as ligands for NK cell inhibitory receptors, such as killer cell immunoglobulin-like receptors (KIRs) in humans and C-type lectin-like Ly49 receptors in mice, thus preventing NK cell activation (12–14). In contrast, cancer cells frequently downregulate MHCI molecule expression which makes them more susceptible to NK cell cytotoxicity, a process referred to as “missing-self” recognition (15). In addition, they increase the production of stress molecules recognized by activating receptors on NK cells. The combination of reduced inhibitory signals (e.g., due to low MHCI expression) and enhanced activating signal (e.g., due to the upregulation stress-induced molecules) effectively triggers the activation of NK cells. Beyond their cytotoxic function, NK cells fulfill a crucial role in modulating adaptive immune responses by releasing cytokines and chemokines and physically interacting with other immune cells, particularly dendritic cells (DCs) (16, 17). Harnessing the potent cytotoxicity and immunomodulatory functions of NK cells has emerged as a promising therapeutic strategy to treat cancer, and readers are referred to recent reviews on the subject (5, 18–20).

Unlike NK cells, CTLs need to be primed by DCs in lymph nodes, before they start to proliferate, acquire an effector phenotype, traffic to and infiltrate the tumor, and recognize and kill cancer cells (21). The specificity of CTLs for their targets is defined by their T cell receptor (TCR) and its affinity for MHCI molecules loaded with antigenic peptides. Activation of CTLs is triggered by the interaction of the TCR-CD3 complex with MCHI molecule-bound cancer-derived antigen in conjunction with co-stimulatory signals from CD8 and CD28. While being activated through different mechanisms, both NK cells and CTLs establish similar intercellular contacts with cancer cells, referred to as lytic immunological synapses (ISs), whose primary functions are to integrate signaling for activation of the killing machinery and facilitate the execution process itself (22, 23).

The prevalent and best-characterized modalities for eliminating cancer cells are the directed secretion of lytic granules containing pro-apoptotic granzymes and pore-forming perforin, as well as expression of death receptor ligands such as TNF-related apoptosis-inducing ligand (TRAIL) and Fas ligand, which can trigger caspase-dependent apoptosis in target cells expressing the cognate receptors (24–26). The lytic granule pathway is recognized as a highly efficient and rapid killing modality, while the death receptor pathway typically operates at a slower pace primarily due to complex apoptotic signaling cascades. Although these mechanisms exhibit different kinetics, they are not mutually exclusive and can work in synergy. For instance, after a few killing events, CLs can switch from a fast granzyme B-mediated killing to a slow death receptor-mediated killing, thus prolonging their killing capabilities (27).

The formation of an IS plays a crucial role in promoting the directed secretion of lytic granules toward the target cells. Following their convergence to the microtubule organizing center (MTOC) along microtubules, lytic granules undergo polarization towards the presynaptic membrane, thus ensuring their precise positioning for subsequent secretion (28). Upon reaching the secretory domain, lytic granules are secreted into the synaptic cleft, which is a narrow intercellular space. Of note, lytic granule polarization and degranulation are uncoupled events in NKs. While ICAM-1 binding to LFA-1 is sufficient to trigger polarization of granules to the IS (29, 30), additional input derived from other activating receptors is necessary to induce granule secretion (31, 32). By tightly restricting the area of secretion, the synaptic cleft not only enhances the concentration of cytotoxic molecules delivered to the target cell but also minimizes the risk of collateral damage to the surrounding healthy cells. Perforin plays a prominent role in CL-mediated cytotoxicity by creating pores in the target cell membrane, thus leading to a repair response that facilitates granzyme uptake and target cell apoptosis (33–37). Interestingly, a recent study has suggested that effective killing of cancer cells by CTLs might require sequential sublethal hits, a process referred to as “additive cytotoxicity” (38).

The prototypical architecture of the mature IS draws its origins from early investigations on T cell synapses, wherein functionalized lipid bilayers were utilized in place of an antigen presenting cell (APC), enabling high-resolution imaging (39–41). The so-called “bull’s eye” model is characterized by three concentric and functionally distinct domains and has played a pivotal role in understanding IS dynamics, encompassing variations in receptors, signaling molecules, and cytoskeletal organization (42, 43). The innermost domain, known as the central supramolecular activation domain (cSMAC), houses a significant concentration of TCRs and features a loose mesh of actin filaments (AFs) that can only be resolved using super-resolution imaging techniques (44, 45). Adjacent to the cSMAC is the peripheral SMAC (pSMAC), serving as an adhesion domain where the integrin leucocyte function-associated antigen-1 (LFA-1) accumulates and interacts with its ligand ICAM-1 on the target cell. The pSMAC’s architecture is characterized by a concentric network of contractile actin arcs assembled by formins and myosin II (46, 47). Finally, the outermost domain, referred to as the distal SMAC (dSMAC), consists of a dense and branched network of actin filaments encircling the periphery of the IS. Detailed investigations of the ISs formed between CTLs or NK cells and partner immune cells or target diseased cells have revealed a rich array of SMAC-based architectures, such as the multifocal ISs, which deviate from the monocentric topology, expanding our understanding beyond the traditional bull’s eye model (22, 48–54). While this observation may not be universally applicable to all models, an intravital microscopy-based comparative study has highlighted that, *in vivo*, NK cells tend to form shorter and more dynamic interactions with tumor cells, whereas CTLs establish longer-lasting contacts (55). Intriguingly, both types CLs exhibited similarly effectiveness in killing their targets, suggesting that the disparities in their interaction dynamics with target cells are likely attributable to differences in their activation mechanism and associated signaling pathways, in particular calcium influx, rather than variations in their ability to induce cell death.

Here, we begin by briefly summarizing the roles of the actin cytoskeleton at the pre-synaptic (CL) side of the IS. We then delve into a more comprehensive review of research studies that explore the organization and dynamics of the actin cytoskeleton at the post-synaptic side of the IS with a primary emphasis on cancer cells. Finally, we discuss the potential impact of actin cytoskeleton remodeling within cancer cells on the overall outcome of CL-mediated cytotoxicity.

## The actin cytoskeleton at the pre-synaptic side of the CL-cancer cell IS

Extensive research spanning several decades has shed light on the diverse functions of the actin cytoskeleton and actin-generated forces at the pre-synaptic side of NK cell and CTL ISs, including initiation, organization, and maintenance of the IS; assembly of signaling complexes and patterning of receptors; activation of receptors; and controlling the exocytosis of lytic granules (22, 43, 56–59). One of the earliest and most significant events triggered by TCR engagement is the formation of a ring-shaped branched F-actin network at the cell-cell interface’s edge (dSMAC). This plays a critical role in facilitating T cell adhesion to the target cell surface (60–62). The signaling pathway involved in this process includes phosphoinositide 3-kinase (PI3K)-mediated generation of phosphatidylinositol-3,4,5-triphosphate (PIP_3_), which subsequently recruits DOCK2, an activator of the Rho family small GTPase Rac1. Consequently, activated Rac1 leads to the activation of WAVE2 at the plasma membrane, which in turn mediates activation of the ARP2/3 complex and actin polymerization. The organization and dynamics of this radial dendritic actin network are reminiscent of those found in the lamellipodium of migrating cells and give rise to a centripetal flow of actin (also referred to as retrograde actin flow), which is essential for assembling the TCR signaling complexes and transporting these complexes to the cSMAC where they undergo recycling through actin-dependent endocytosis (63–67). The centripetal flow of actin also directly contributes to stimulating T cell activation by providing the mechanical force required for optimal TCR signaling and promoting integrin conformational change (68–73). In turn, integrin engagement constrains the centripetal flow of actin by recruiting focal adhesion proteins such as talin and vinculin and paxillin, thus modulating the motion of signaling complexes and TCR signaling (74). A similar centripetal flow of actin driving adhesion molecules and receptors toward the IS center exists in the NK cell IS. Its velocity is modulated by the activity of ARP2/3 complex regulator Wiskott-Aldrich-Syndrome-Protein (WASp) and myosin IIA (75) and critically determines the conformation and activity of SH2 domain-containing protein tyrosine phosphatase-1 (SHP-1) - a key inhibitory regulator of the NK cell response (76–78).

The establishment of the synaptic secretory domain involves a reduction in cortical F-actin density at the IS center, which leads to the formation of a permissive network (22, 28, 44, 45, 79–82). Additionally, nanoscale actin dynamism, mediated by the ARP2/3 complex and myosin IIA, generates mechanical forces that facilitate degranulation (79, 83). In CTLs, rapid recovery of higher cortical F-actin levels follows granule exocytosis and directs termination of secretion, potentially by acting as a mechanical barrier (83, 84). Prior to their secretion into the synaptic cleft, lytic granules converge toward the MTOC and subsequently polarize along with the MTOC to the secretory domain (22, 85). The processes of granule convergence and MTOC repositioning to the IS are complex and remain not fully understood (86), with dynein, a microtubule minus-end-directed motor protein, being implicated in these processes (87, 88). Recent evidence highlights the critical role of cortical clearance of F-actin within the IS in dynein recruitment and MTOC reorientation, thus illustrating that the actin and tubulin cytoskeletons closely interact to coordinate CL polarization and exocytosis (86, 89, 90). Intriguingly, the F-actin cross-linking protein FLNa has been identified to facilitate multiple steps of NK cell cytotoxicity, including conjugation to target cells, synaptic accumulation of F-actin, and lytic granule degranulation, while limiting the production of IFN-γ and TNF-α (91). This suggests that modulation of actin dynamics contributes in steering CLs toward cytotoxicity or regulatory functions through cytokine production. Readers are referred to recent reviews for a more comprehensive description of the regulation and roles of the actin cytoskeleton at the pre-synaptic side of the IS (57, 90, 92, 93).

## Actin dynamics at the post-synaptic side of the immunological synapse: lessons from lymphocyte-dendritic cell interactions

Until recently, the exploration of actin dynamics in cancer cells during their interaction with CLs and formation of the lytic IS has been limited. However, studies on other types of ISs formed by T cells and NK cells have shed light on the importance of actin dynamics in the conjugated target cells particularly dendritic cells (DCs) in regulating IS formation, organization, and activity. For a more comprehensive discussion on the actin cytoskeleton at the DC side of the IS, readers are invited to refer to a recent review that provides information beyond the scope of the present section (94). Over two decades ago, pioneering investigations unveiled the active reorganization of the actin cytoskeleton in DCs during their interaction with resting CD4+ T cells, thus leading to a robust polarization of F-actin and actin regulatory proteins, such as the actin bundling protein fascin, towards the IS (95–97). Such remodeling of the DC actin cytoskeleton is necessary for optimal cell-cell conjugation and activation of T cells.

Additional evidence of the changes in actin dynamics occurring in DCs during conjugation with T cells was provided by fluorescence recovery after photobleaching (FRAP) analysis, which revealed an overall stabilization of filamentous actin at the cell-cell interface compared to distal cortical regions (98). More recent studies show the mechanistic details underlying the role of actin cytoskeleton remodeling in DCs in the regulation of the T cell IS (99). Remarkably, depolymerization of F-actin in DCs induces the conversion of the T cell IS from a multifocal to monofocal topology (49, 99), thus supporting the involvement of the DC actin cytoskeleton in patterning the T cell IS. Similar to the T cell side of the IS, the DC side exhibits a multifocal topology that is characterized by multiple and dynamic small and larger actin patches separated by actin hypodense areas. The inhibition of the lateral movement of these patches following the genetic ablation of the Wiskott-Aldrich-Syndrome-Protein (WASP)-family verprolin-homologous protein (WAVE) regulatory complex (WRC), a critical regulator of the ARP2/3 complex, leads to prolonged cell-cell conjugation time and reduction in T cell priming efficiency (99). Such effects are primarily attributed to an overstabilization of ICAM-1 on the DC surface through the activation of ERM proteins that connect ICAM-1 and the cortical actin cytoskeleton, and the subsequent increase in synaptic ICAM-1-LFA-1 interactions and overstabilization of the IS.

As previously stated, the mechanical forces exerted by the actin cytoskeleton play critical role in modulating TCR signaling. For an in-depth understanding of this role and the underlying molecular mechanisms, readers are referred to an excellent review article (43). While the mechanical forces applied to the TCR stem from the actin-dependent pushing and pulling forces exerted by CLs on the APC surface (100, 101), they are also contingent upon the biomechanical properties of the APC - particularly the cortical stiffness, which is mostly governed by the cytoskeleton (99, 102, 103). Remarkably, DCs undergo a 2-3-fold (actin cytoskeleton-dependent) increase in cortical stiffness during their maturation, which is perceived by T cells as a co-stimulatory signal (104). As part of the underlying mechanism, cortical stiffening increases TCR complex mechanical stimulation, thus leading to enhanced downstream signaling and lowering the antigenic threshold required to activate T cells (43, 100, 101, 103–106). Of note, both the ARP2/3 complex- and formin-mediated actin polymerization contribute to the increased cortical stiffness of mature DCs (104).

Similar to T cells, NK cells physically interact with DCs *via* an IS. NK cells contribute to the selection of immunogenic DCs by selectively killing immature DCs. This editing process is critical for the initiation of anti-cancer immune responses by promoting successful T cell priming (107, 108). However, NK cells also engage with mature DCs within a non-lytic IS, thus leading to a reciprocal cross-talk between the two cell types, which is crucial for orchestrating and regulating the immune response against cancer. Such, non-lytic, NK-DC IS is referred to as the regulatory IS (109) and facilitates the acquisition of effector functions by naïve NK cells (110–113). In turn, DC-activated NK cells can stimulate DC maturation (114). The investigation into how mature DCs evade destruction while synapsing with NK cells has shed light on the key role of the DC actin cytoskeleton in facilitating this process (115). Throughout the conjugation process, there is a gradual relocation of the filamentous actin cytoskeleton in DCs towards the synaptic region. Such cytoskeletal polarization was associated with a blockade of NK cell cytotoxicity as indicated by the absence of MTOC and perforin polarization to the IS. Remarkably, genetic or pharmacological inhibition of actin polymerization in DCs induced the conversion of the regulatory IS into a lytic IS as indicated by NK cell MTOC and perforin polarization, as well as a significant increase in degranulation, IFN-γ production, and DC killing. As an underlying mechanism, DC-derived F-actin was shown to induce the polarization of MHCI molecules to the IS and restrict their diffusion away from the IS.

Collectively, these studies emphasize the crucial role of the synaptic actin cytoskeleton in DCs, which plays a pivotal role in modulating the organization of T cell and NK cell ISs and regulating the synaptic density of signaling molecules, thus ultimately safeguarding DCs from CL-mediated cytotoxicity. Additionally, emerging evidence suggests that the cortical stiffness of DCs, which is primarily determined by cytoskeletal dynamics, acts as a critical regulator of the mechanical response of T cells. These findings imply that specific configurations or characteristics of the cortical actin cytoskeleton of cancer cells may be indispensable for facilitating efficient CL-mediated killing. Cancer cells may exploit actin cytoskeleton remodeling to alter the morphology and activity of the lytic IS and evade immune destruction. These intriguing possibilities will be evaluated and discussed below.

## Intrinsic cytoskeletal properties of cancer cells and the lytic immunological synapse

Compelling evidence suggest that mechanical forces within the lytic IS significantly influence CL cytotoxicity potential through multiple mechanisms (43, 116). As previously discussed, these forces play a crucial role as co-activating signals in both CTLs and NK cells, thus enhancing immunoreceptor affinity for ligands and promoting conformational changes in receptors and integrins, which subsequently amplify downstream signaling (117–121). Moreover, CL-originating mechanical forces have been shown to potentiate the pore-forming activity of perforin by increasing target cell membrane tension, thus enhancing target cell killing (122, 123). In turn, recent research has highlighted the significance of target cell rigidity as a pivotal factor governing the magnitude of the synaptic forces, as well as its impacts on lytic IS formation and activity (124). For instance, experiments utilizing stiff beads that were functionalized with antibodies against the NK activating receptor NKp30 and LFA-1 have proven highly effective in triggering NK cell degranulation (116). Conversely, softer beads fail to induce the NK cell’s MTOC and lytic granule polarization, and only lead to the formation of poorly stable IS.

The implications of target cell rigidity are especially pertinent to cancer because cancer cells tend to undergo softening during the metastatic cascade and the acquisition of invasive properties, primarily due to alterations in their cytoskeletal architecture (125–130). In this regard, a recent study established a compelling link between the mechanical softness of cancer cells and their ability to hinder the formation of membrane pores by perforin. The data suggested that this evasion mechanism is utilized by a subset of undifferentiated, stem cell-like, cancer cells (characterized by their intrinsic softness) to escape CTL-mediated cytotoxicity (131). The manipulation of cellular stiffness using pharmacological or genetic approaches targeting the actin cytoskeleton was proven to dictate the susceptibility of both murine cancer cell models and primary human cancer cells to perforin-mediated perforation. Most remarkably, stiffening tumor cells with the actin polymerization promoting drug jasplakinolide significantly improved antitumor T cell immunity and PD-1 blockade immunotherapy in *in vivo* mouse models. Softening tumor cells with the myosin II inhibitor blebbistatin had opposite effects. Another important finding of this study is the identification of MYH9 - the heavy chain of non-muscle myosin IIA that crosslinks and contracts actin filaments to produce mechanical forces (132) - as a critical molecule for perforin pore formation. A MYH9 Knockout proved sufficient to make cells softer and inhibit the pore-forming activity of perforin in several cancer cell models. Although the exact mechanism remains elusive, perforin might interact with MYH9 and thus trigger an opposite force whose strength is proportional to the amount of F-actin linked to MYH9. While this force would be sufficient to induce perforin active conformation in the case of stiff cancer cells, it would remain insufficient in the case of soft cells.

The association between the actin polymerization status of cancer cells and their susceptibility to CL-mediated lysis was previously suggested in an earlier study that focused on a variant of a non-small-cell lung carcinoma cell line exposed to *in vitro* CTL selection pressure (133). This particular resistant variant exhibited a substantial reduction in filamentous actin content, resulting in a consistent shift toward a more rounded cellular morphology. Transcriptomics uncovered notable dysregulation in the expression of various genes. Among those, two genes encoding cytoskeleton regulators, ephrin-A1 and scinderin, were markedly upregulated and played a pivotal role in driving alterations in cell morphology alterations and conferring resistance to CTL-mediated lysis. Although target cell perforation was not specifically evaluated in this model, it is noteworthy that the resistant cells were proficient in inducing CTL degranulation. This observation raises the possibility that these cells may exhibit resistance to pore-forming activity. Another intriguing observation was the disorganization of the IS formed between CTLs and resistant cells, characterized by a more loosely organized synaptic cleft and a reduced number of close contacts when compared to the IS formed with susceptible cells.

Another recent study has unveiled an important link between the expression levels of myocardin-related transcription factors (MRTFs), the rigidity of cancer cells and the control of tumors by CLs (134). MRTFs play a pivotal role as co-factors for the serum response factor (SRF), responding to actin polymerization by dissociating from actin monomers and relocating to the nucleus where they transcriptionally activate a range of SRF-target genes related to the actin cytoskeleton (135). MRTFs are indispensable for actin-based cell migration during physiological processes and cancer progression (136). As cancer cells disseminate *via* the bloodstream and infiltrate supportive perivascular niches, their ability to establish prominent metastatic lesions relies on their adhesion and spreading onto the endothelial basement membrane (137). This process critically requires MRTF activity (138, 139). Strikingly, Tello-Lafoz et al. have established that beyond their pro-metastatic function in facilitating adaptation to the metastatic niche, MRTFs paradoxically sensitize cancer cells *in vivo* to CL-mediated destruction, and increase the effectiveness of PD1 and CTLA4 checkpoint blockade therapies (134). Mechanistic investigations have indicated that MRTF-induced actin polymerization increases the stiffness of cancer cells, which stimulates IS formation and CL cytotoxic potential as demonstrated by increased levels of cytokine secretion and degranulation.

Further strengthening the concept that cancer cell stiffness is an actionable therapeutic point of intervention, membrane cholesterol depletion-induced stiffening of cancer cells has recently been demonstrated to enhance CTL-mediated cytotoxicity as well as the efficacy of adoptive T-cell therapy in preclinical mouse models of solid tumors (140). The vulnerability of cancer cells resulting from membrane cholesterol depletion primarily depends on actin cytoskeleton-dependent forces exerted by CTLs at the IS. It is noteworthy that, in contrast to the cytoskeleton rigidification resulting from MRTF overexpression (134), cholesterol depletion fails to induce a significant increase in CTL cytokine production or degranulation (140). This intriguing disparity underscores distinctions in underlying mechanisms among the approaches targeting the enhancement of cancer cell rigidity. Additional investigations are required to unravel and characterize these intricate mechanistic distinctions.

## Actin dynamics at cancer cell side of the immunological synapse

The target cell side of the lytic IS has long been regarded as a relatively passive area and is primarily characterized by the array of molecules present on its surface. This perception has mainly evolved from the historical focus on the presynaptic cell side of the IS as well as the use of reductionists target cell models amenable to high-resolution microscopy such as glass-supported planar lipid bilayers wherein specific proteins are embedded to initiate IS formation. However, a growing body of research centered around cell-cell conjugates has underscored the dynamic nature of the cancer cell side - particularly with regards to actin dynamics and vesicular and membrane trafficking (57, 141–143).

In a recent study, we thoroughly investigated the distribution of the actin cytoskeleton within breast cancer cells upon encountering NK cells. We found that a subset of breast cancer cells responded to the attack of NK cells by exhibiting a prominent accumulation of filamentous actin within the synaptic region (144). This intriguing response somehow paralleled the cytoskeletal modifications observed in DCs during their interaction with NK cells in the context of the regulatory synapse (115, 145). Moreover, akin to DCs, the synaptic accumulation of actin filaments was associated with breast cancer cell resistance to NK cell-mediated cytotoxicity. Remarkably, such resistance could be reverted by silencing CDC42 or N-WASP, two regulators of the ARP2/3 complex, thus leading to a return of cancer cells to a susceptible phenotype (144). In a follow-up study we extended the concept of actin remodeling-mediated resistance to several chronic lymphocytic leukemia (CLL) cell lines and primary cells from CLL patients (146). Inhibition of synaptic actin accumulation using a CDC42-targeting drug - combined with an anti-HLA-G blocking antibody as a mean to increase conjugate formation - resulted in a substantial enhancement of NK cell-mediated killing of primary CLL cells.

The precise mechanism through which the polarization of cancer cells’ actin cytoskeleton to the IS leads to resistance to NK cell-mediated cytotoxicity remains unclear. The accumulation of F-actin in the postsynaptic region of the IS has been linked to diminished levels of granzyme B and a reduction in apoptosis within target cells (144, 146). However, whether this reduction stems from actin cytoskeleton-mediated resistance against perforin, the degradation of granzyme B, or inhibition of NK cell killing activity requires further investigation. Remarkably, similar to the scenario observed in the DC-NK cell inhibitory IS (115), breast cancer cells exhibiting synaptic F-actin accumulation also display increased levels of HLA-A,-B,-C and PD-L1 at the IS (144). This observation suggests that F-actin accumulation at the postsynaptic side of the IS might act as a scaffold for the clustering of NK cell inhibitory ligands, leading to potent inhibitory signal against NK cells. This remains largely speculative and will require rigorous experimental evaluation.

The mechanism responsible for initiating actin remodeling in NK cell-conjugated cancer cells remains a subject of ongoing investigation. Interestingly, K562 cells, characterized by their lack of MHCI expression and highly susceptibility to NK cell-meditated cytotoxicity have been documented as incapable of remodeling their actin cytoskeleton in response to NK cell conjugation (115). This intriguing observation raises the possibility that MHCI expression might be instrumental in driving the polarization of F-actin to the post-synaptic side of the IS. It is also unclear why discrete cells within the same cancer cell line do not uniformly exhibit the same ability to respond to NK cell attack by actin cytoskeleton remodeling (144). This variability may arise from the inherent phenotypic heterogeneity within a single cell line including factors such as the epithelial-mesenchymal transition (EMT) status of individual cells (147). This diversity can contribute to differing capacities to promptly reconfigure the actin cytoskeleton in response to NK cell IS formation. Notably, the process of EMT has been recognized as a critical driver of tumor immune evasion (148), and has been implicated in the overall proficiency of cancer cell lines to effectively polarize their actin cytoskeleton towards the IS (144).

## Concluding remarks

Recent breakthroughs have prompted an increasing interest in actin dynamics at the cancer cell side of the IS and support the notion that the actin cytoskeleton of cancer cells is a double-edged sword exerting a pivotal influence on the regulation of CL cytotoxic activity. The cytoskeletal and biophysical properties of cancer cells are exploited by CLs to adjust their response to the biochemical signals present at the surface of cancer cells - a mechanism termed mechanosurveillance. Notably, the stiffening of the actomyosin cytoskeletal network, e.g., during cancer cell spread and colonization at new metastatic sites, serves as a trigger for the assembly of the IS and subsequent unleashing of lytic activity. This cytoskeleton-mediated rigidification of cancer cells amplifies force-dependent signaling in CLs, thus enhancing their proficiency in target cell recognition. Furthermore, elevated mechanical tension stands indispensable for the assembly of perforin-based transmembrane pores in cancer cells, which are pivotal for mediating cytotoxic effects.

Conversely, the softening of the actin cytoskeleton - a phenomenon often accompanying the transition of cancer cells towards an invasive and metastatic state - has emerged as a novel immune checkpoint exploited by cancer cells to dampen CL cytotoxicity. For a more comprehensive discussion on the synergistic interplay between biomechanical and biochemical cues leading to a robust antitumor immune response, readers are encouraged to refer to the recent review article “Harder, better, faster, stronger: biochemistry and biophysics in the immunosurveillance concert” (124).

Our live cell investigations into actin dynamics within cancer cells during their interaction with NK cells have revealed the remarkable ability of cancer cells to rapidly and extensively remodel their actin cytoskeleton upon engaging with NK cells, regardless of gene expression changes. This cytoskeletal remodeling is characterized by massive polarization of filamentous actin towards the IS, thus mirroring the cytoskeletal changes occurring in CLs during the IS initiation. The polarization of the cancer cell’s actin cytoskeleton provides robust protection against CL-mediated killing by efficiently reducing intracellular granzyme B levels within target cells. Nevertheless, the elucidation of the precise underlying mechanism requires further investigation. F-actin accumulation at the tumor cell side of the IS could be considered as an evasion strategy per se, possibly through inducing IS disorganization and destabilization, or as a fundamental cellular process shared across multiple synaptic evasion tactics. This remains an open question. While this remains speculative, augmenting synaptic actin dynamics could facilitate the recruitment of lysosomes and subsequent membrane repair (143), stimulate autophagy-mediated degradation of granzyme B (149), promote the synaptic recruitment of inhibitory ligands and immune checkpoint molecules (115, 144), and trigger local changes in membrane composition mediating increased resistance to perforin (145, 150). A concise summary of the primary findings and hypotheses discussed in this review article is provided in **Table 1**.

**Table 1:** Key findings and hypotheses regarding the impact of actin cytoskeleton remodeling within cancer cells on the lytic immunological synapse.

Actin cytoskeleton changes	Effects on the immunological synapse	Underlying mechanisms	Cytoskeletal effector proteins	Associated genetic pathways	Overall outcome	Progression stage	Refs.
**Stiffening of the cortical actin cytoskeleton**	▪ Stimulation of synapse formation ▪ Enhanced CL activation (degranulation, cytokine secretion) ▪ Enhanced target cell perforation	▪ Cell rigidification ▪ Mechanical stimulation of CLs ▪ Enhanced receptor signaling ▪ Enhanced perforin pore-forming activity	▪ ARP2/3 ▪ CDC42 ▪ Formins	▪ Upregulation of SRF target actin cytoskeleton genes promoted by actin polymerization-induced nuclear translocation of MRTFs	▪ Increased cytotoxicity ▪ Reduced metastasis ▪ Increased survival ▪ Improved response to ICB	▪ Colonization of the metastatic site	▪ (43) ▪ (124) **▪ (134)** ▪ (140)
**Softening of the cortical cytoskeleton**	▪ Attenuated target cell perforation ▪ Morphological alteration of the synapse	▪ Cell softening ▪ Insufficient mechanical tension to induce perforin active conformation ▪ Reduced MYH9-mediated force generation due to limited amounts of F-actin	▪ MYH9	▪ Downregulation of MYH9 expression ▪ upregulation of scindrin and ephrin-A1	▪ Reduced cytotoxicity ▪ Faster tumor growth and increased tumor incidence ▪ Reduced response to immunotherapy	▪ Tumor-repopulating cells ▪ Acquisition of migratory/ invasive properties ▪ Metastatic dormancy?	▪ (116) ▪ (124) **▪ (131)** ▪ (133)
**Actin cytoskeleton polarization toward the immunological synapse**	▪ Reduced levels of GzB and apoptosis within target cells ▪ Synapse destabilization? ▪ Reduced CL activation?	▪ Synaptic polarization of inhibitory and immune checkpoint molecules	▪ N-WASP ▪ CDC42	▪ Fast response to interaction with CLs; does not require a change in gene expression	▪ Resistance to CL-mediated cytotoxicity	▪ Association with the epithelial-mesenchymal transition (EMT) process	▪ (57) ▪ (115) **▪ (144)** ▪ (146) ▪ (150)

The last column provides important references, with the primary research articles of the highest significance highlighted in bold.

Innovating therapeutic approaches aiming to enhance physical contact between CLs and cancer cells or APCs and increase CL cytotoxicity against cancer cells, hold tremendous potential in cancer treatment (151). In particular, chimeric antigen receptors (CAR)-T and CAR-NK cell therapies have shown great promise – particularly in hematologic malignancies (152–160). Preclinical models and advanced intravital imaging techniques have been combined to provide real-time visualization of cancer cell elimination by CAR-CLs at the single-cell level within living organisms (161–164). Such breakthrough technologies open exciting avenues for in-depth mechanistic investigations and for translating these findings into clinical applications, such as the prospective assessment of the clinical effectiveness of CAR-based therapies. CAR synapses display several structural and functional differences compared to conventional ISs (53, 165), thus potentially limiting the effectiveness of CAR-based therapies. Recent advancements such as the incorporation of an intracellular scaffolding protein binding site, a PDZ binding domain, to the CAR of CAR-T or -NK cells, have shown improved IS formation and CL polarization, leading in turn to enhanced anti-tumor activity and prolonged survival in several *in vivo* tumor models including in solid tumors (166). The development of various CAR products has catalyzed the expansion of clinical trials aimed at addressing a wide spectrum of hematologic and solid malignancies (167). In the light of the present discussion, the concept of targeting the postsynaptic actin cytoskeleton, in conjunction with optimized CAR CLs, or alternative CL redirecting strategies, holds significant potential for triggering a potent anti-tumor response. The combination of CAR-based therapy with other treatment modalities, such as radiotherapy or immune checkpoint blockade, has emerged as one of the most auspicious approaches and is being currently under evaluation in clinical trials (168, 169). However, the ubiquitous expression of the actin cytoskeleton and its multiple roles in physiological processes - particularly within immune responses - pose significant challenges for the development of therapeutic approaches aimed at manipulating the organization and dynamics of the actin cytoskeleton within cancer cells. Furthermore, unlike microtubule-targeting drugs that exhibit a preference for selectively impairing rapidly dividing cancer cells and have demonstrated efficacy in treating various malignancies, actin-targeting drugs result in unacceptable levels of toxicity precluding their clinical utility. The identification of relevant molecular targets will require a thorough dissection of the cancer cell side of the IS and the development of innovative methodologies that can distinguish between the pre- and post-synaptic sides of the IS (170).

## Author contributions

CT: Conceptualization, Funding acquisition, Supervision, Writing – original draft. EO: Conceptualization, Writing – original draft. LF: Supervision, Writing – review & editing. DPF: Writing – review & editing. CH: Writing – review & editing.
